# The Imperative Need of Metal Salt for the Treatment of Industrial Wastewater via the Synergic Coagulation-Flocculation Method

**DOI:** 10.3390/polym14091651

**Published:** 2022-04-20

**Authors:** Bader S. Al-Anzi, Mehraj-ud-din Naik, Mudasir Ahmad

**Affiliations:** 1 Department of Environmental Technology and Management, Kuwait University, P.O. Box 5969, Safat 13060, Kuwait; 2 Department of Chemical Engineering, College of Engineering, Jazan University, Jazan 45142, Saudi Arabia; mnaik@jazanu.edu.sa; 3 School of Chemistry and Chemical Engineering, Northwestern Polytechnical University, Xi’an 710072, China; mirmudasirv@gmail.com

**Keywords:** Kuwait tile industry, wastewater, jar test, coagulation, ferric chloride, turbidity

## Abstract

Tile industry wastewater is known to contain a high concentration of TSS and turbidity resulting from various raw materials. In the present study, the effectiveness of the coagulation process on turbidity and TSS removal from Kuwait ceramic tile industry wastewater was investigated using ferric chloride as a coagulant. The experiments were conducted using jar tests to determine the optimum operating conditions of coagulant dosages, pH, and settling time. It was found that the coagulant dosage and medium pH greatly affect the efficiency of the coagulation process. A gradual increase in coagulant dosage from 10 to 50 mg/L increased the efficiency of turbidity removal from 95.6% to 99.5%. The efficiency of the coagulation process was also found to be dependent on pH values, where higher pH improved the efficiency of turbidity removal. It was found that a medium pH of 10, 1 h settling time, and 50 mg/L of coagulant dosage are the optimum process conditions to achieve almost complete removal of turbidity (99.5%) and TSS (99.8%). This study concluded that coagulation might be useful as a primary wastewater treatment process for tile industry wastewater.

## 1. Introduction

Water management is a progressively critical issue in most conventional industrial areas that causes water resource pollution and halts sustainable development [1,2,3]. The industrial wastewater produced from different industries consists of hazardous contaminants like suspended solids, heavy metals, cyanides, refractory organics, and dissolved inorganics, which causes risks to human health and creates pollution of the environment, soil, and water bodies [4,5]. Meanwhile, the inadequate treatment of wastewater represents a costly exercise [4]. The ceramic tile industry is one of the most popular industries globally. It utilizes large amounts of groundwater resources like fresh water, thus causing a scarcity of groundwater resources [6]. The manufacture of conventional materials like tiles and ceramics in the ceramic industry produces ceramic wastes and wastewater, which contains toxic pollutants [3,7,8] that affect plant tissues and the human body [9,10]. Therefore, it is necessary to treat the wastewater before it enters the water bodies. A lot of work will implement waste recovery systems and methods to recycle ceramic wastes [11].

The wastewater from the industry is treated with different methods such as chemical treatment, chemical precipitation, and biological treatment. This has been adopted to remove the suspended solids, dissolved organic compounds, and metals ions present [12]. Most Middle Eastern countries, including Kuwait, are characterized by severe arid climatic conditions and have a very limited annual rainfall of about 110 mm/yr [13]. Kuwait has one of the highest per capita water consumption rates and the lowest level of renewable international freshwater resources per capita in the world [14,15]. Kuwait’s rapid infrastructure development and population growth are expected to exert severe pressure on the limited groundwater reserves. In this context, the potential reuse of treated wastewater is considered an important element of water resource development and management [16]. The ceramic and tile manufacturing industry typically requires a large quantity of energy while producing both liquid and solid wastes [17]. Several ceramic and tile factories in Kuwait manufacture various types of tiles for different needs, such as residential and commercial purposes [18]. The wastewater is discharged untreated to the environment, thus posing a serious pollution threat to the soil and water in Kuwait. Therefore, the environmental management of the tile industry in Kuwait is becoming crucial, and the tile factories are requested to treat their effluent before discharging it into the environment. Ironically, little attention has been paid to treating ceramic industry wastewater worldwide, and limited studies have been reported in the literature. These reported studies also focused mainly on treating organics through biological processes [19]. Studies on the removal of solids and associated turbidity from the ceramic tiles industry have rarely been reported. The situation is grimmer concerning Kuwait as no information on the quantity and quality of wastewater discharged from these industries exists. Studies on possible treatment options for ceramic and tile industry wastewater generated in Kuwait are absent. The present study is probably the first attempt to conduct a detailed analysis of the wastewater generated from the tile industry in Kuwait. Moreover, the application of a physicochemical process in the treatment of tile industry wastewater has been reported. 

The present work chose the industrial wastewater generated from a Kuwait tile factory located in the Sabhan district for the study. A physicochemical treatment consisting of coagulation-flocculation was applied to find the effectiveness of FeCl_3_. The coagulation-flocculation process is regarded as one of the most important and widely used treatment processes of industrial wastewater due to its simplicity and effectiveness [20,21]. This process does not only remove turbidity from the water but also other contaminants such as fine particulate matters, bacteria, and colors. The efficiency of the coagulation-flocculation process is highly dependent on the medium pH, coagulant dosage, and settling time [22,23]. Therefore, the present study demonstrates the implementation of a sustainable physicochemical process to reduce the pollution load of the tile industry on the environment and illustrates an effective and innovative approach that can be effectively implemented in similar industries worldwide.

## 2. Materials and Methods

### 2.1. Kuwait Tile Wastewater Sample

FeCl_3_ was purchased from Merck. A wastewater sample was obtained from a tile manufacturing company located in the Sabhan district of Kuwait. This tile manufacturing company generates about 4000 gallons of wastewater per day. The wastewater was analyzed for its physiochemical characteristics such as TSS, TDS, pH, conductivity, turbidity, alkalinity, chloride, sulfate, hardness, Ca^2+^, Mg^2+^, Fe, Cu, COD, and BOD using standard analytical methods. The results of the analysis are provided in Table 1.

### 2.2. Batch Experiment

A jar test was used to find the effect of coagulant dosage, pH, and settling time. Jar tests were carried out under the batch mode of operation, accommodating six beakers with paddles. The jars were placed in an even manner such that the contents prepared in different coagulant dosages present in the beakers would be mixed thoroughly. The low/high switch controlled the stirring speed of the paddles. The procedure was conducted by filling beakers with 1 L of the wastewater to be treated after measuring pH, turbidity, TDS, TSS, etc. The pH value of the individual beakers was adjusted, and coagulant (FeCl_3_.6H_2_O) was added to each beaker with concentrations of 10, 20, 30, 40, and 50 mg/L. The wastewater in the beakers was mixed at a high speed (100 rpm) for 1 min, followed by a lower speed (40 rpm) for 15 min. The process was stopped for 1 h to allow flocs to precipitate and separate from the water. Samples were collected from the supernatant and analyzed to measure the effectiveness of each coagulant dosage. The jar test tested the addition of coagulants to the actual wastewater effluent samples. Coagulation is different from flocculation as follows:

❖ Coagulation precedes flocculation;
❖ Chemicals (coagulants) are added in the coagulation step;
❖ Mixing in coagulation is fast and occurs for a short time (seconds up to a minute);
❖ In flocculation, no chemicals are added, with gentle mixing occurring for a long time (minutes).

We used the same beaker to conduct coagulation, flocculation, and sedimentation in the current work. First, we added the chemicals with high-speed mixing for a short time (coagulation), then conducted gentle mixing for a long time (flocculation), and finally, we allowed settling to occur without any mixing for about an hour (sedimentation).

**Figure 1:**
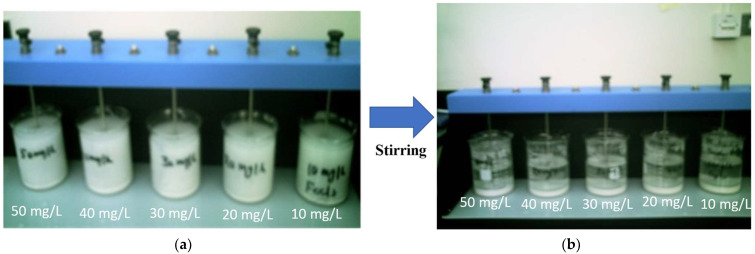
Photos of beakers with (**a**) untreated and (**b**) treated industrial wastewater.

To find the optimum pH for conducting a jar test, experiments were conducted using 1L of wastewater sample, and FeCl_3_ was used as a coagulant. The experiments were conducted by varying the pH values of 6, 7, 8, 9, and 10 while keeping other variables (coagulant concentration of 50 mg/L and settling time of 1h) constant. The effect of settling time on turbidity reduction was conducted using 1L of wastewater samples. The experiments were conducted by adding a 50 mg/L of coagulant at different settling time intervals of 30, 60, 120, and 180 min to determine the optimum settling time. The effectiveness was determined by the reduction in the turbidity of wastewater samples at each dosage parameter. Figure 1 shows the wastewater samples before and after the addition of the coagulant.

## 3. Results and Discussion

### 3.1. Characteristics of Kuwait Tile Factory-Industrial Wastewater

Values of various parameters of Kuwait tile factory industrial wastewater were analyzed using FeCl_3_, and the various parameters of the tile factory wastewater are listed in Table 1. The physicochemical characteristics of the wastewater shown in the present study suggest that it is highly contaminated with inorganics (e.g., Ca^++^, Cl^−^, TSS, TDS, conductivity, and alkalinity) (Table 1) and thus violates the standard proposed by KEPA for industrial discharge to sewer [24]. Results showed that the concentrations of TSS, TDS, alkalinity, pH, hardness, Ca^++^, and Cl^−^ were significantly higher than the acceptable limit set by KEPA, while COD, BOD, Mg, and SO_4_ concentrations were within the acceptable ranges. In Table 1, it is evident that the wastewater stream’s TSS and turbidity concentration were almost 90 times and 270 times higher than what is permissible by KEPA, respectively. The wastewater also consists of colloidal solids that are usually metal precipitates and are minute particles lesser than 1 micron that do not settle easily due to the presence of such high colloidal and suspended solids, reducing the clarity of the water. Hence, its removal is very important in the treatment of wastewater. The presence of suspended solids and colloidal particles that show high turbidity in ceramic and tile industry wastewater has been reported earlier [25,26]. Coagulants are added to industrial effluents to help in removing suspended particles. A research study conducted by Sagar et al. for a typical ceramic industry indicated that the performance of ferric chloride on the removal of turbidity was better than that of alum [6]. The turbidity removal consistently decreased at different dosages of ferric chloride compared to alum. In another study, the effectiveness of aluminum sulfate and ferric chloride was evaluated by Akbar et al. [27]. Results indicated that the ferric chloride coagulant showed higher turbidity removal efficiency of 92.9 to 99.4% at optimum conditions, particularly for highly turbid waters. Inorganic coagulants like FeCl_3_ produce large volumes of floc and entrap bacteria while settling compared to polymer coagulants, which produce smaller volumes of floc. Thus, when inorganic coagulants are used appropriately, they can remove suspended solids more effectively [28,29]. As observed in Table 1, the pH of the wastewater from the Kuwait tile factory industry is 12.8 and is considered above the permissible limits of KEPA. FeCl_3_ operates well over a wide range of pH [27,28]. When FeCl_3_ is added to water, it hydrolyzes, consuming alkalinity, and thus has a significant effect on pH. However, coagulants like alum or Polyaluminum chloride (PACl) are pretreated during their manufacture and thus do not show a significant impact on the pH and alkalinity [30]. The promising performance in removing suspended solids, better accessibility, and cost-effectiveness of FeCl_3_ made it a better choice as a coagulant in the present study.

### 3.2. Effect of Dosage

**Figure 2:**
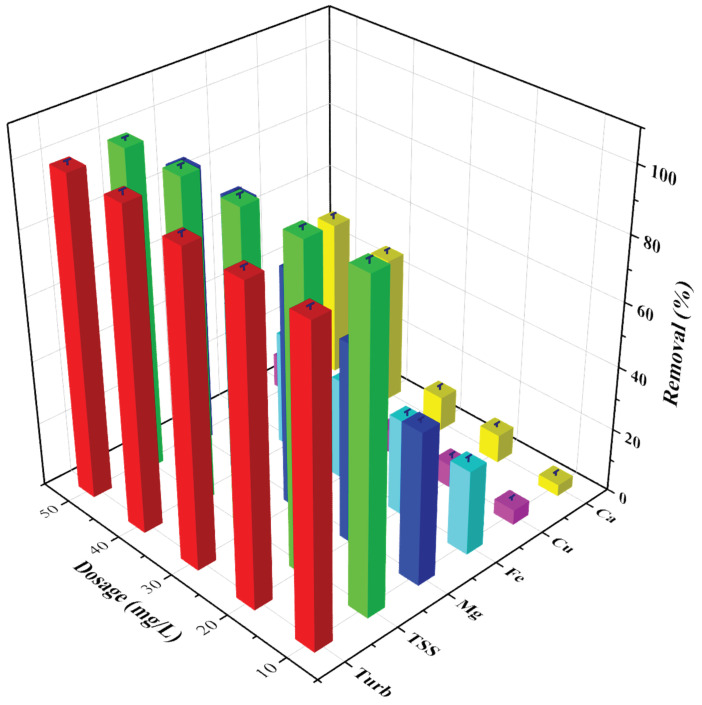
Removal efficiency of FeCl_3_ under various experimental conditions in the Kuwait tile industry wastewater (dosage = 10 to 50 mg/L, pH = 10 and t = 1 h) (error bars represent ± error, n = 3).

The residual turbidities and TSS of the wastewater at different coagulant dosages (10–50 mg/L) measured after the completion of tests are provided in Figure 2. As observed in Figure 2, the residual turbidity of the supernatant water decreased gradually with increased coagulant concentrations, and the residual NTU was observed as 70 NTU at a 50 mg/L coagulant dosage. This shows that a high concentration of coagulant favors turbidity removal. It can be observed that 99.5% of turbidity was removed at a coagulant dosage of 50 mg/L compared to 95.6% at 10 mg/L dosages. This can be explained by the fact that at higher coagulation dosages, an increase in the possibility of particle-particle collision during mixing exists, leading to higher turbidity removal. Generally, it is recognized that in adsorption by polymers, the solute compotator and chelators in solution affect the metal ion removal. The competition of ions in wastewater limits the removal of metal ions by polymers by blocking the active sites present on the polymer backbone [31]. Iron salts are rapidly hydrolyzed in water to give cationic species, which can be absorbed by negatively charged suspended particles and neutralize their charge. During the reaction, particles are destabilized so that flocculation can occur and lead to the removal of turbidity. The results obtained in this study agree with an earlier study conducted with water which suggested that high coagulant concentration favors coagulation efficiency [32,33]. It should be noted that a further increase in the coagulant dosage would have caused overdosing, which in turn can cause restabilization of the colloid particles [34]. Additionally, since the turbidity removal was almost stable at dosages greater than 30 mg/L, it was not used above 50 mg/L. As a result of turbidity reduction, a significant reduction in the TSS was also observed at all coagulant concentrations, though residual TSS was lowest at the highest coagulant concentrations. About 99.79% of the TSS was removed at 10 mg/L of coagulant concentration (Figure 2), whereas it increased marginally to 99.85% at 50 mg/L of coagulant dosage. It is observed that only 0.06% enhancement is observed when the coagulant dosage was increased from 10 mg/L to 50 mg/L and, therefore, a further increase in the coagulant dosage would not bring a further significant effect to the percentage removal and is not economically feasible. Thus, 50 mg/L was chosen as the optimum coagulant concentration for efficient removal of turbidity and TSS in the study. 

### 3.3. Effect of pH

**Figure 3:**
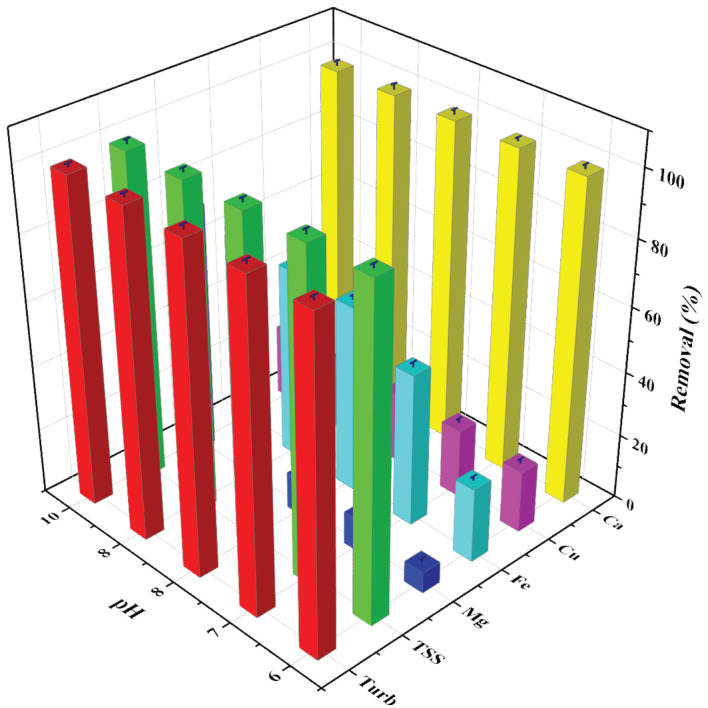
Removal efficiency of FeCl_3_ under various experimental conditions in Kuwait tile wastewater (pH = 6 to 10, coagulant dosage = 50 mg/L and t = 1 h) (error bars represent ± error, n = 3).

The residual turbidities and TSS of the wastewater at different pH values (pH 6 to 10) measured after the sedimentation tests are provided in Figure 3. The experiments on the effect of pH were not carried out at pH values lower than 6 due to the solubilization of natural tile powders in an acidic medium. As observed in Figure 3, the residual turbidity of the supernatant water at a medium pH of 6 was 108 NTU, whereas it decreased gradually with an increase in pH to 10 (15 NTU). This showed that higher pH favors coagulation, and about 99.8% of the turbidity was removed at pH 10. 

Generally, FeCl_3_ works according to the equation:

$$2{\mathrm{FeCl}}_{3}+6H_{2}O+\mathrm{Ca}{\left({\mathrm{HCO}}_{3}\right)}_{2}\to 3{\mathrm{CaCl}}_{2}+2\mathrm{Fe}{\left(\mathrm{OH}\right)}_{3}+6{\mathrm{CO}}_{2}+12H_{2}O$$

The insoluble $\mathrm{Fe}{\left(\mathrm{OH}\right)}_{3}$ acts as gelatinous flocs that settle through the wastewater and sweep out the suspended material in the wastewater. 

### 3.4. Effect of Time

**Figure 4:**
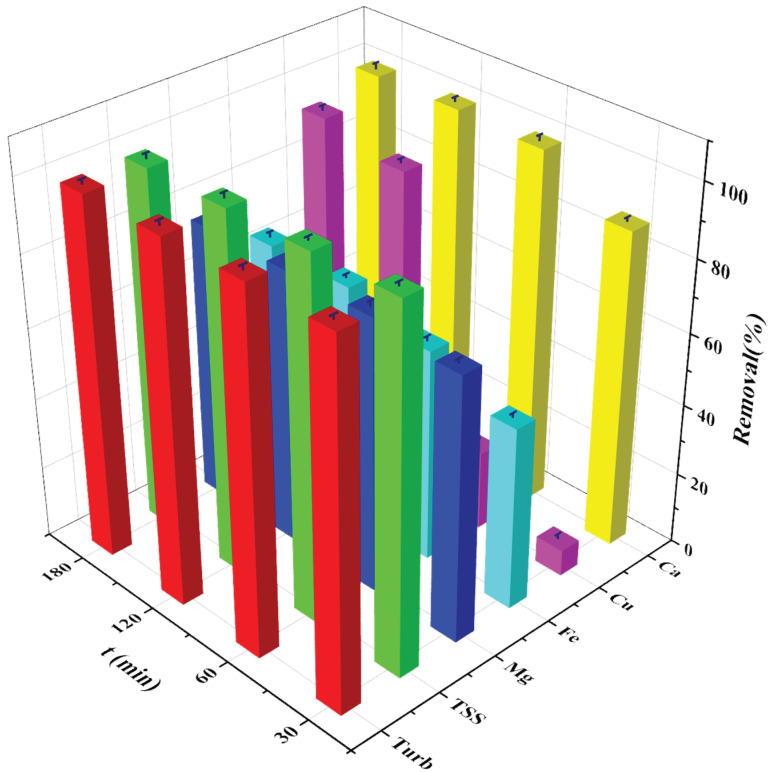
Removal efficiency of FeCl_3_ under various experimental conditions in Kuwait tile wastewater (t = 30 to 180 min, coagulant dosage = 50 mg/L and pH = 10) (error bars represent ± error, n = 3).

The turbidity and TSS removal were increased with increasing time, though the removal was found to be at its maximum at the highest settling time. The residual turbidity of the supernatant water after the 30 min settling period was about 180 NTU, but it decreased to about 15.5 at 1 h. Further increases in time beyond 1 h showed only a slight decrease in residual turbidity at 2 h and 3 h, with 10 NTU and 5.8 NTU, respectively (Figure 4). This shows that a 1 h time is sufficient to remove the complete turbidity from the tile wastewater. Similarly, about 98.9% of the TSS was removed within 30 min, and almost 99.93% of the TSS was removed within the 1 h settling period. The removal of TSS increased further marginally to 99.96% at 3 h. The present study results agree with the reported range of between 30 min and 120 min [35,36,37,38,39]. As the concentration of suspended solids in the raw wastewater was considerably high, colloidal and suspended particles destabilized due to the adsorption of strongly charged partially hydrolyzed ions at a longer settling time. According to Koohestanian et al., most of the suspended solids in water possess a negative charge, and the surface also has a similar charge. Since similar charges repel each other when they are in contact, the particles remain in suspension instead of combining and settling down faster [40]. The addition of coagulants neutralizes the negative charges on non-settleable solids. Thus, the destabilized suspended particles upon collision enable the formation of small flocs, which grow into denser aggregates. Sometimes the micro flocs are not quite visible, and the water might appear clear. Thus, a longer duration of settling time will enable neutralization of all the charged particles present in the water, thus improving the efficiency of turbidity and TSS removal. The present study agrees with a study performed by James et al., which confirmed that removal efficiencies were higher over a longer period [30]. Studies carried out by Muhammad et al. also indicated that the use of FeCl_3_ showed enhanced turbidity removal upon a prolonged settling period of 3 h compared to other coagulants [41].

## 4. Conclusions

In this work, a metal salt was used to analyze the feasibility of the coagulation process in removing the TSS and turbidity from Kuwait tile industry wastewater. It was found that the coagulation process engaged FeCl_3_ as an effective way to remove turbidity from highly contaminated wastewater. The results suggested that the coagulant dosage, medium pH, and time significantly affect the removal efficiency of the coagulation process. The optimal dosage was 50 g/L, with a removal efficacy for turbidity and TSS of 99.5% and 99.9% from industrial wastewater. Overall, 1 h time, pH 10, and 50 mg/L of coagulant dosage were the optimum conditions at which complete removal of turbidity and TSS (99.8%) can be observed. Our study confirmed that using FeCl_3_ is an efficient and economical method for use as an effective primary treatment process for high-turbidity industry wastewater.

## Figures and Tables

**Table 1:** Physiochemical properties of wastewater sample.

Tile Industry Wastewater					KEPA Standards for Industrial Discharge to Sewer (Parson 2002)		
Parameters	pH	(mg/L)	Turbidity (NTU)	Conductivity (µS/cm)	pH	Concentration (mg/L)	Turbidity (NTU)
TSS	12.8	26,350	13,610	7975	6-8	300	50
TDS		4800				1500	
COD		22.9				750	
BOD		0.27				500	
Alkalinity		3600				--	
Chloride		1203				--	
Hardness		119.4				--	
Ca		44.9				--	
Mg		1.77				--	

## Data Availability

The data presented in this study are available on request from the corresponding author.

## Abbreviations

TSS	Total suspended solids
FeCl_3_	Ferric chloride hexahydrate
TDS	Total dissolved solids
COD	Chemical oxygen demand
BOD	Biological oxygen demand
Ca	Calcium
Mg	Magnesium
Fe	Iron
Cu	Copper
KEPA	Kuwait Environmental Public Authority
SO_4_	Sulfate
PACl	Polyaluminum chloride
NTU	Nephelometric Turbidity Units
Fe(OH)_3_	Ferric hydroxide
